# Three-Dimensional Stochastic Off-Lattice Model of Binding Chemistry in Crowded Environments

**DOI:** 10.1371/journal.pone.0030131

**Published:** 2012-01-17

**Authors:** Byoungkoo Lee, Philip R. LeDuc, Russell Schwartz

**Affiliations:** 1 Department of Biological Sciences and Lane Center for Computational Biology, Carnegie Mellon University, Pittsburgh, Pennsylvania, United States of America; 2 Departments of Mechanical and Biomedical Engineering, and Lane Center for Computational Biology, Carnegie Mellon University, Pittsburgh, Pennsylvania, United States of America; Aston University, United Kingdom

## Abstract

Molecular crowding is one of the characteristic features of the intracellular environment, defined by a dense mixture of varying kinds of proteins and other molecules. Interaction with these molecules significantly alters the rates and equilibria of chemical reactions in the crowded environment. Numerous fundamental activities of a living cell are strongly influenced by the crowding effect, such as protein folding, protein assembly and disassembly, enzyme activity, and signal transduction. Quantitatively predicting how crowding will affect any particular process is, however, a very challenging problem because many physical and chemical parameters act synergistically in ways that defy easy analysis. To build a more realistic model for this problem, we extend a prior stochastic off-lattice model from two-dimensional (2D) to three-dimensional (3D) space and examine how the 3D results compare to those found in 2D. We show that both models exhibit qualitatively similar crowding effects and similar parameter dependence, particularly with respect to a set of parameters previously shown to act linearly on total reaction equilibrium. There are quantitative differences between 2D and 3D models, although with a generally gradual nonlinear interpolation as a system is extended from 2D to 3D. However, the additional freedom of movement allowed to particles as thickness of the simulation box increases can produce significant quantitative change as a system moves from 2D to 3D. Simulation results over broader parameter ranges further show that the impact of molecular crowding is highly dependent on the specific reaction system examined.

## Introduction

Chemistry in a living cell operates very differently than would be predicted from models of the same chemical reactions in an idealized *in vitro* environment, which is diluted and well-mixed [1]. Many features of a living cell make the intracellular environment distinctive, such as compartmentalization, active transport, the cytoskeleton network, and molecular crowding. More accurately addressing the effects on molecular interactions of these key features of cellular reaction systems is crucial to building more realistic models of reaction systems in the *in vivo* environment. Molecular crowding – i.e., the dense crowding of many kinds of macromolecules in a cell – directly influences many fundamental biological processes, such as protein folding [2], [3], protein aggregation and assembly [4]–[7], enzyme activity [8], [9], reaction kinetics [10], [11], and signal transduction [12]. Molecular crowding can hinder diffusion and provide strong steric hindrance to various reaction types, either inhibiting or enhancing chemical reactions based on many parameters of the system in question [13]–[15]. These complicated interrelated parameters make the strength and direction of the crowding effect extremely hard to accurately predict for any given model system.

Previously, we developed a two-dimensional stochastic off-lattice model (2DSOLM) [16] based on Green's function reaction dynamics [17]. The model was designed to better satisfy two constraints that confront all computational models: realism and efficiency. For example, continuum models such as ordinary differential equations and lattice Monte Carlo models [18]–[20] are very efficient but greatly simplify the actual system being modeled. Coarse-grained particle models, such as Brownian dynamics models using hard sphere particles with simplified interaction potentials [21], provide greater accuracy in exchange for increased computational cost. Full atomic resolution particle models [22], [23] provide even more realistic models of particles' behavior but at a high computational cost that makes it infeasible to simulate large systems or long time scales, especially in highly crowded conditions. Our prior stochastic off-lattice model (SOLM) uses Green's function reaction dynamics (GFRD) [17] to simulate realistic Brownian particle trajectories with reduced computational cost using discrete event models. In addition, SOLM uses a simple coarse-grained particle model to allow one to vary multiple parameters relevant to the crowding effect without the need for detailed and costly atomic structure calculations. Other similar approaches have proven successful for modeling reaction chemistry in crowded conditions. The virtual cytoplasm method also relies on a particle-based off-lattice model on a similar mesoscopic scale to address molecular crowding, but uses fixed time and space steps rather than the fully continuous time and space allowed by GFRD [24]. Kim and Yethiraj's reaction model uses Brownian dynamics combined with coarse-graining to similarly simulate the effect of crowding on a model of membrane receptor reactions [25]. In addition, Tsao, Minton, and Dokholyan's didactic model compares an analytical with a simulation method to uncover how the crowding effect alters protein folding and association using a toy model [26].

We have further shown that it is possible to use such simplified particle simulations to train multiparameter regression models, providing a method for predicting the effects of crowding on reaction systems that can combine the fast runtime of simple analytical methods with the greater versatility of particle models [27]. This simulation-based approach provides a more general and efficient algorithm to build a stochastic reaction model in various crowded conditions that can be expected to be easily extensible to more complicated models of particle structure and dynamics for which analytical models are unsuited. In addition, our stochastic models can easily investigate the crowding effect for different physical and system parameters by allowing us to alter the parameter value singly or in combination. In the present work, we extend our SOLM model from 2D to 3D, while retaining the GFRD and coarse-graining approach key to our model's efficiency, and compare the two model variants. Two-dimensional models have shown considerable value for exploring the theory of crowding, given their simplicity and relative computational tractability, but the question remains whether conclusions drawn from such models are of significant value in describing three-dimensional system. The question is particularly significant for “nearly” two-dimensional systems, such as diffusion in a membrane or at the leading edge of a migrating cell, where two-dimensional models have extra appeal. We specifically examine whether the parameter dependencies of crowding observed in our prior 2D models are qualitatively the same as those in 3D and how the quantitative behaviors vary as we interpolate between the two. This study is intended to help judge when one can rely on conclusions from 2D models and how well the two dimensional particle models and associated regression approach will extend to 3D systems. The work provides guidance for the degree to which we can rely on prior 2D models as descriptions of generic crowding phenomena and where 2D or 3D models can be trusted in modeling either 3D or pseudo-2D systems.

## Results

### Crowding simulations

**Figure 1:**
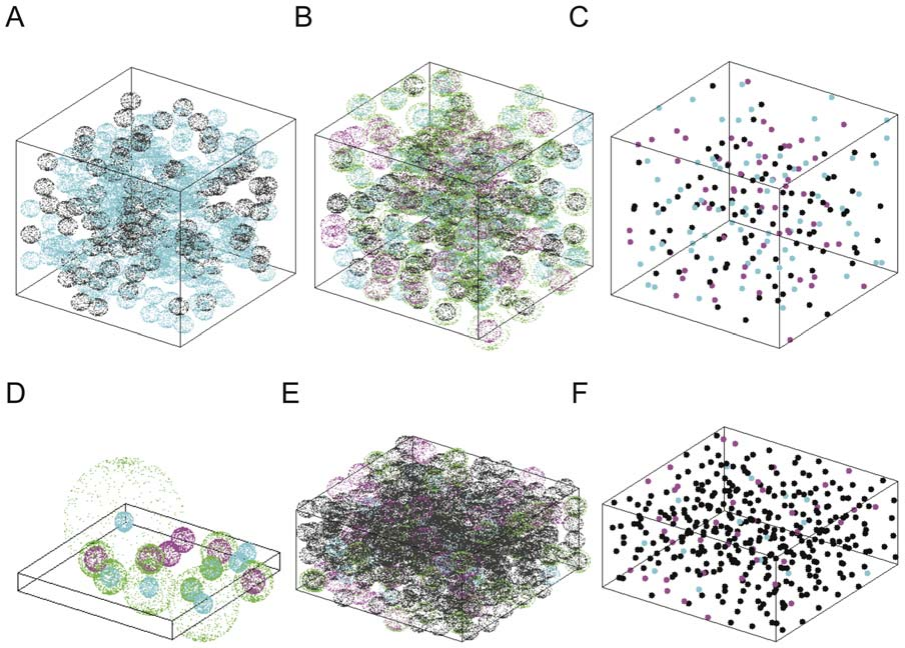
Simulation snapshots of 3DSOLM. (A) 0.1*C_R_*+0.05*C_I_* in a 50 nm×50 nm×50 nm space at the initial state, (B) 0.1*C_R_*+0.05*C_I_* in a 50 nm×50 nm×50 nm space at the quasi-equilibrium state (25 µs), (C) the same condition as (B) but showing only the center position of each particle to aid the visualization, (D) 0.1*C_R_* in a 50 nm×50 nm×5.125 nm space at the quasi-equilibrium state (25 µs), (E) 0.1*C_R_*+0.35*C_I_* in a 50 nm×50 nm×25.625 nm space at the quasi-equilibrium state (25 µs), (F) the same condition as (E) but showing only the center position of each particle to aid the visualization. Cyan spheres represent reactant monomers, magenta spheres represent reactant dimers, black spheres represent inert crowding agents, and outer green spheres represent diffusion limit spheres for (A), (B), (D), and (E). (C) and (F) use the same color scheme for the center positions of the particles.

We characterized the effects of crowding on reaction chemistry across model types by examining the effects on a simple homodimerization test system for a variety of parameter sets. We examined two different homodimerization test cases for investigating the crowding effect on the model binding system: one using a varying reactant concentration (*C_R_*: measured by the volume ratio of occupied reactant particles to the simulation box) without any inert crowding agent and the other using a fixed reactant concentration with additional varying inert crowding agent concentration (*C_I_*: measured by the volume ratio of occupied inert crowding particles to the simulation box). For these two test cases, we used a 50 nm×50 nm×50 nm cubic simulation space and used default parameter values, explained in Methods. We simulated eight *C* values (0.1, 0.15, 0.2, 0.25, 0.3, 0.35, 0.4, 0.45: measured by the volume ratio of occupied particles to the simulation box) for varying reactant concentrations without any inert crowding agents. For the second test case, we fixed reactant concentration to 0.1 and changed the inert crowding agent concentrations (*C_I_*) for eight *C* (*C_R_*+*C_I_*) values (0.1+0.0, 0.1+0.05, 0.1+0.1, 0.1+0.15, 0.1+0.2, 0.1+0.25, 0.1+0.3, 0.1+0.35). Figure 1 illustrates 3DSOLM simulation. Figure 1(A) and (B) show simulation snapshots of the initial state and the quasi-equilibrium state (25 µs) of the 0.1*C_R_*+0.05*C_I_* test case, respectively. Figure 1(C) shows the center positions of particles in Fig. 1(B), for better visualization. Cyan spheres represent reactant monomers, magenta spheres represent reactant dimers, black spheres represent inert crowding agents, and green spheres represent diffusion limit spheres, a construct of the GFRD algorithm describing the volume in which a particle might have diffused with appreciable probability since its position was last evaluated.

**Figure 2:**
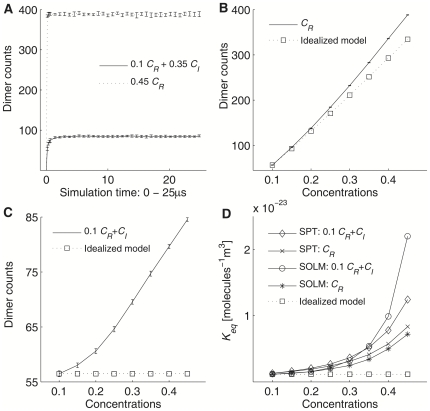
Crowding simulation results. (A) Reaction progress for 0.1 *C_R_*+0.35 *C_I_* and 0.45 *C_R_* without inert crowding agents, with *C* = 0.45 for both test cases, (B) Dimer counts from 3DSOLM and the idealized model for the pure *C_R_* (0.1, 0.15, …, 0.45), (C) Dimer counts from 3DSOLM and the idealized model for the 0.1 *C_R_*+ additional *C_I_* (0.0, 0.05, …, 0.35), (D) *K_eq_* from 3DSOLM, the idealized model, and SPT for the 0.1 *C_R_*+ additional *C_I_*, pure *C_R_* (0.1, 0.15, …, 0.45). The simulation space is 50×50×50 nm^3^.

Figure 2 shows simulation results for these two test cases. Figure 2(A) shows the reaction progress of a homodimerization reaction from 0 to 25 µs for both 0.1 *C_R_*+0.35 *C_I_* and 0.45 *C_R_* without any inert crowding agent. The reaction progress curve shows the number of dimers versus time for 10 independent simulation runs. The curve starts at zero because all reactants are initially monomers, and then quickly moves to the quasi-equilibrium state within 2 µs for the most crowded case (*C* = 0.45) with default parameter values. After 2 µs, it fluctuates around the average value with a seemingly consistent range for the remainder of the simulation due to random exchanges between monomers and dimers after the model reaction reaches its equilibrium state. Because the less crowded cases reach quasi-equilibrium faster, we assume that 5 µs is sufficient time to reach quasi-equilibrium for the test reaction system and this 5 µs interval is a reasonable upper bound on mixing time across crowding levels in our simulation conditions. We examine a total of 5 time points (5, 10, 15, 20, 25 µs) for each simulation run for analyzing simulation results in order to measure the long term behavior of the test reaction system after quasi-equilibrium based on our assumption of the upper bound on mixing time. To better display the rapid changes early in the simulation, the plot shows a resolution of 0.15625 µs for the first five time points then averages over 0.78125 µs intervals for subsequent time points. Figure 2(B) shows the number of dimers for pure *C_R_* simulations at the quasi-equilibriums state. Comparing with the idealized model, which is calculated based on an idealized mass-action model of Eq. (21) in the Methods using simulation data at *C* = 0.1, the average number of dimers increases 1.16 fold from 0.1 to 0.45 *C_R_*. Even without any inert crowding agent, the reaction can still be influenced by crowding from reactant molecules themselves. Figure 2(C) shows the number of dimers for fixed 0.1 *C_R_*+additional *C_I_*. The average number of dimers increases up to 1.5-fold as the concentration of inert crowding agents increases from 0.0 *C_I_* to 0.35 *C_I_*, and it clearly shows a strong crowding effect compared to the idealized model, again calculated based on an idealized mass-action model using simulation data at *C* = 0.1. Estimated *K_eq_* values, calculated using Eq. (19) in Methods and shown in figure 2(D), demonstrate more clearly how the crowding effect alters the reactions. The *K_eq_* curve is dramatically increased by adding either additional inert crowding agents or reactants. Inert crowding agents cannot change their excluded volume through binding, as reactants can, and so they provide a stronger steric hindrance and a stronger crowding effect than reactants alone for given parameter conditions and initial concentration.

We also calculated the estimated *K_eq_* using statistical thermodynamics and scaled particle theory (SPT) [14], [28], [29] by assuming that the 1% pure reactant case was reasonably diluted condition so that non-ideal interactions among particles were negligible. Based on this assumption, we ran additional simulations for 1% pure reactant case, and used as the results of these low-concentration simulations to represent the reaction in the ideal state, explained in detail in Methods. These additional SPT data show how the particle simulations can deviate from expectations from an analytical model explicitly accounting for the excluded volume effect of macromolecular crowding. The estimation from SPT showed similar enhancement of *K_eq_* as the total concentration increased. As with SOLM, the 0.1 *C_R_*+additional *C_I_* cases showed a stronger crowding effect than the pure *C_R_* case in SPT. However, the difference between SOLM and SPT increased at densely crowded conditions (*C* = 0.4–0.45) for the 0.1 *C_R_*+additional *C_I_* cases, while the pure *C_R_* case did not. This observation suggests that the particle simulations reveal effects of crowding beyond purely total excluded volume, an issue examined in the next section.

### Parameter variations

A major concern of our approach is how parameter changes in 3DSOLM affect binding chemistry, singly or in combination, for various crowded conditions. Here, we examine the following physical parameters of the homodimerization simulations: the probability of binding upon collision between two reactant monomers; the mean time between dissociation events, defined as the inverse of the dissociation rate constant; the diffusion coefficient for reactants and inert crowding particles; and the volume ratio of dimer to monomer, reflecting the relative compactness of a dimer. To simulate the various parameter values, we used a 50 nm×50 nm×50 nm cubic simulation box and tested a fixed reactant concentration with varying inert crowding agent concentrations. We simulated five binding probability values (*B* = 0.1, 0.3, 0.5, 0.7, 0.9), five breaking mean time values (*M* = 0.6, 0.8, 1.0, 1.2, 1.4 ns), five diffusion coefficient values (*D* = 1.3, 4.63, 7.97, 11.3, 14.63×10^−11^ m^2^s^−1^), and five ratios of dimer to monomer volume (*α* = 1.6, 1.8, 2.0, 2.2, 2.4). For each simulation, we simulated eight total concentration values (*C* = *C_R_*+*C_I_* = 0.1+0.0, 0.1+0.05, 0.1+0.1, 0.1+0.15, 0.1+0.2, 0.1+0.25, 0.1+0.3, 0.1+0.35). For each such test, all parameter values aside from the one being tested were held at their default values: *B* = 0.7, *M* = 1.0 ns, *D* = 4.63×10^−11^ m^2^s^−1^, *α* = 2.0, *β* = 1.0, *d_th_* = 0.125 nm.

**Figure 3:**
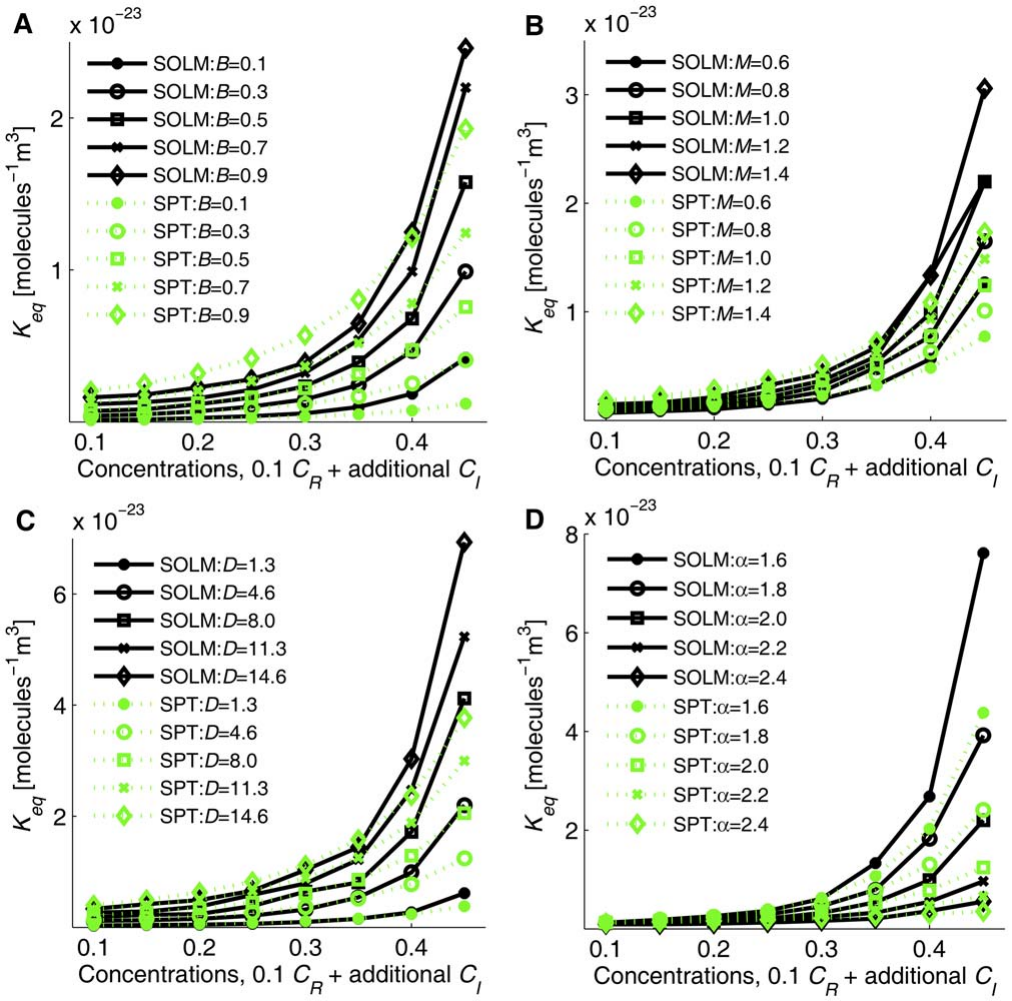
*K_eq_* estimated from SOLM and SPT for fixed 0.1 *C_R_*+ additional *C_I_* and varying parameter values (*B*, *M*, *D*, *α*). (A) Variation across five *B* values (0.1 bottom, 0.3, 0.5, 0.7, 0.9 top), (B) Variation across five *M* values (0.6 bottom, 0.8, 1.0, 1.2, 1.4 ns top), (C) Variation across five *D* values (1.3 bottom, 4.63, 7.97, 11.3, 14.63×10^−11^ m^2^s^−1^ top), (D) Variation across five *α* values (1.6 top, 1.8, 2.0, 2.2, 2.4 bottom). All other parameter values for each test are set to their default values: *B* = 0.7, *M* = 1.0 ns, *D* = 4.63×10^−11^ m^2^s^−1^, *α* = 2.0, *β* = 1.0, and *d_th_* = 0.125 nm in a 50 nm×50 nm×50 nm simulation box.

Figure 3 shows *K_eq_* values of SOLM and SPT for these four varying parameters (*B*, *M*, *D*, and *α*), calculated by simulation data and Eq. (19) and 1% simulation data and Eq. (22), respectively. Several features are apparent in the figure. First, all *K_eq_* curves for both SOLM and SPT show increasing *K_eq_* with increasing concentrations of inert crowding particles. Second, the equilibrium state shows a noticeable response to all parameter variations. In detail, increasing *B*, *M*, or *D* or decreasing *α* increase the equilibrium constant and thus produce more dimers, based on *K_eq_* values from both SOLM and SPT. *K_eq_* values estimated by SPT match well with those from SOLM for low (*C* = 0.1) to moderate levels of crowding (*C* = 0.3). However, SPT starts to appreciably underestimate *K_eq_* as estimated from simulations starting at a moderate level of crowding, and the difference between the two increases as the total concentration increases, similar to figure 2(D). SPT is based on statistical thermodynamic calculations of the volume exclusion effect to derive corrections to the equilibrium constant at the ideal state. The 1% concentration simulations using SOLM provide reasonably accurate *K_eq_* values for the ideal condition, based on correction factors in table 1. Thus SPT values closely follow those of the simulations in the range of low to middle concentrations. However, the difference between SPT and SOLM tends to increase at higher total concentrations. We attribute this difference primarily to two factors. First, SPT estimates crowding effects based on a model of pairwise interactions of excluded volume that neglects the relatively higher excluded volume effect implied when one considers the maximal possible packing densities of a lattice of spheres [28], while SOLM accounts for this effect by explicitly modeling the individual particles in a simulation. SOLM, on the other hand, may overstate the crowding effect at the high end because of its use of a threshold distance (*d_th_*) beyond a particle's physical radius at which interactions between particles can occur. Particles are required to be outside this distance of one another after reaction or collision events, effectively causing an increase in the crowding level.

**Table 1:** *K^o^* [1×10^−25^molecules^−1^m^3^] and Г_exc_ for various parameter conditions.

*B*	*K^o^*	Г_exc_	*M*	*K^o^*	Г_exc_	*D*	*K^o^*	Г_exc_	*α*	*K^o^*	Г_exc_	*H*	*K^o^*	Г_exc_
0.1	0.85	1.05	0.6	5.77	1.05	1.3	2.79	1.05	1.6	9.80	1.04	5.125	12.48	1.04
0.3	2.98	1.05	0.8	7.59	1.04	4.6	9.39	1.04	1.8	9.88	1.04	10.25	10.83	1.04
0.5	5.63	1.05	1.0	9.39	1.04	8.0	15.70	1.04	2.0	9.39	1.04	15.375	10.33	1.04
0.7	9.39	1.04	1.2	11.27	1.04	11.3	23.09	1.04	2.2	9.27	1.04	20.5	10.13	1.04
0.9	14.69	1.04	1.4	13.19	1.04	14.6	29.18	1.04	2.4	9.29	1.04	25.625	9.91	1.04

*K^o^* values are calculated by 100 independent simulation runs of 3DSOLM for the 1% pure reactant case in 100 nm×100 nm×100 nm for varying *B*, *M*, *D*, and *α* cases and 400 nm×400 nm×Height (*H* nm) for different heights of simulation boxes. Except for the specific parameter examined in each experiment, all parameter values are set to the default values: *B* = 0.7, *M* = 1.0 ns, *D* = 4.6×10^−11^ m^2^s^−1^, *α* = 2.0, *β* = 1.0, *d_th_* = 0.125 nm in 3DSOLM.

One of the difficulties in accurately modeling crowded systems is the complex patterns of cross-dependency between distinct parameters. We chose to examine simultaneous changes in the *α* and *C*, parameters shown in 2D to exhibit cross-correlated non-linear effects on binding equilibria in crowded media [27]. Figure 3(D) shows a similar cross-dependency between *α* and *C* in 3D. A smaller *α* in highly crowded conditions exaggerates the crowding effect relative to that seen with larger *α* while the crowding effect is small across the range of *α* values examined at low to moderate levels of crowding.

**Figure 4:**
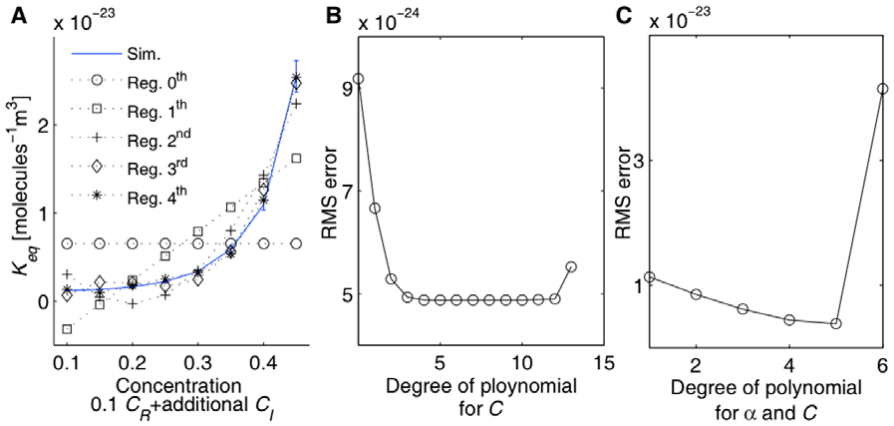
Leave-one-out cross validation test to determine the best fitted regression model. (A) Simulation curve for fixed 0.1 *C_R_*+ additional *C_I_* with all other parameters set to the default values and best fit regression curves for different degree of polynomials, (B) Root mean square error values for the leave-one-out cross validation (5 time points for each run, 10 independent runs for 25 µs), (C) Root mean square error values for the leave-one-out cross validation for simultaneous variation in *α* and C (five *α* values:1.6, 1.8, 2.0, 2.2, 2.4) and (eight *C* values: 0.1, 0.15, …, 0.45).

To more quantitatively analyze the different parameter effects on equilibria for various crowded conditions in 2D and 3D, we built regression models for each parameter case. We first built a regression model for 3D using least-squares fitting. Figure 4(A) shows *K_eq_* curves of the simulation and best fit regression models for varying degrees of polynomial from 0^th^ to 4^th^ order. We chose a fourth degree polynomial based on leave-one-out cross validation tests, shown in figure 4(B). The regression model of *K_eq_* for the default parameter case is
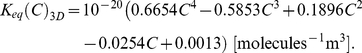
(1)


As shown in the best-fit regression model of Eq (1), the total concentration nonlinearly altered the equilibrium of reaction system. This nonlinear effect of the total concentration parameter on the binding reaction has been observed in previous experiments [4]–[7], [14], [22] and in our previous 2D simulations [27], [30]. To reasonably compare between 2D and 3D, we built an additional regression model for 2D using the same degree as in the 3D case based on previous 2D simulation data [27], [30]. The best-fit regression model in 2D is given in Eq. (2).
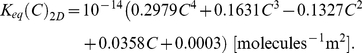
(2)


Note that, because of the difference in simulation space in 2D (100 nm×100 nm) and 3D (50 nm×50 nm×50 nm) and the resulting different units of concentration, the absolute coefficients of the regression polynomials in 3D and 2D are not directly comparable.

**Figure 5:**
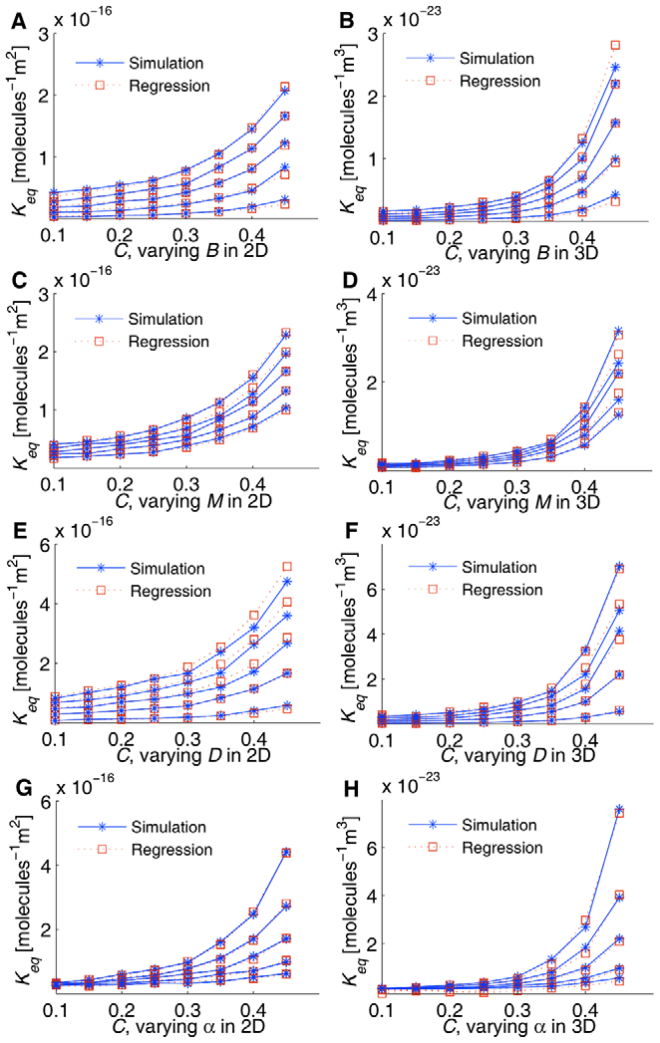
Simulation *vs.* Regression for *K_eq_* in 2D and 3D. (A, B) varying parameter *B* (0.1 bottom, 0.3, 0.5, 0.7, 0.9 top), (C, D) varying parameter *M* (0.6 bottom, 0.8, 1.0, 1.2, 1.4 ns top), (E, F) varying parameter *D* (1.95 bottom, 6.95, 11.95, 16.95, 21.95×10^−11^ m^2^s^−1^ top) for 2D and (1.3 bottom, 4.63, 7.97, 11.3, 14.63×10^−11^ m^2^s^−1^ top) for 3D cases, (G, H) varying parameter *α* (1.6 top, 1.8, 2.0, 2.2, 2.4 bottom). The first column (A,C,E,G) shows 2D cases and the second column (B,D,F,H) shows 3D cases. Simulation curves show averages from 10 independent runs for 3D and 30 independent runs for 2D at 5 time points (5, 10, 15, 20, 25 µs) per run for fixed *C_R_* = 0.1 and varying *C_I_* (0.0, 0.05, 0.1, 0.15, 0.2, 0.25, 0.3, 0.35).

Comparison between best-fit regression models in 2D and 3D, however, shows that the influence of total concentration on crowding is qualitatively similar between 2D and 3D. The other three parameters (*B*, *M*, and *D*) show a similar effect in 2D and 3D, both quantitatively and qualitatively. The parameters *B*, *M*, and *D* separately and linearly influence the equilibrium state of the model reaction system for both 2D and 3D. Figure 5(A), (C), (E), and (G) show the *K_eq_* curves of simulation and best-fit regression models for 2D and figure 5(B), (D), (F), and (H) show the *K_eq_* curves of simulation and best-fit regression models for 3D for varying *B* (0.1, 0.3, 0.5, 0.7, 0.9), *M* (0.6, 0.8, 1.0, 1.2, 1.4 ns), *D* (1.95, 6.95, 11.95, 16.95, 21.95×10^−11^ m^2^s^−1^ for 2D and 1.30, 4.63, 7.97, 11.30, 14.63×10^−11^ m^2^s^−1^ for 3D), and *α* (1.6, 1.8, 2.0, 2.2, 2.4), with other parameters set to default values (*B* = 0.7, *M* = 1.0 ns, *D* = 6.95×10^−11^ m^2^s^−1^, *α* = 2.0, *β* = 1.0, *d_th_* = 0.5 nm for 2D and *B* = 0.7, *M* = 1.0 ns, *D* = 4.63×10^−11^ m^2^s^−1^, *α* = 2.0, *β* = 1.0, *d_th_* = 0.125 nm for 3D simulations). The best-fit regression models for 2D and 3D in this figure are
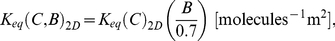
(3)

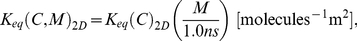
(4)

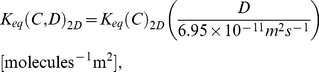
(5)

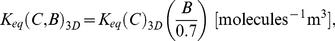
(6)

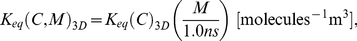
(7)

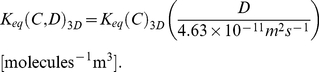
(8)


Thus, binding probability upon collision between two reactants (*B*), mean time of dissociation reaction (*M*), and diffusion coefficient (*D*) independently and linearly altered the equilibrium constants and these parameter effects on *K_eq_* can be accurately predicted by linear scaling for both 2D and 3D cases.

The volume ratio of dimer to monomer (*α*), however, shows a strong cross-dependency with the total concentration parameter (*C*) and must be fit in a multi-dimensional parameter space, similar to 2DSOLM [30]. Figure 4(C) shows the leave-one-out cross validation results for various degree of polynomial of *α* and *C* for 3DSOLM. The fifth-degree polynomial was selected as the best-fit regression model. Using the same polynomial least-square fitting method [30], the regression polynomial of *α* and *C* is
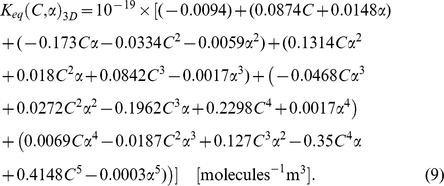



Figure 5(H) shows the *K_eq_* curves from the average values of simulations and fit values from the regression polynomial of Eq. (9) for varying *α*. To aid comparison, we again built regression models for the 2D case to match the degree of the best-fit 3D model, using fifth degree models for *α* and *C* in both 2D and 3D, as shown in Eq. (10).
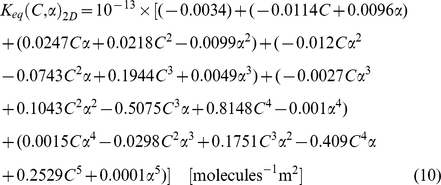



The regression models in 3D and 2D show that the parameter effect of *α* is again nonlinear and cross-dependent with *C*, but can be accurately predicted by a high-order polynomial regression model, as shown in figure 5 (G,H).

### Interpolating between 2D and 3D models

The densely crowded environment can impede diffusion and provide steric hindrance to reaction events for both reactants and products of a chemical reaction. The impact of crowding on diffusion would be expected to act differently in a 2D versus a 3D model, although it is not *prima facie* clear how the extra dimension will specifically alter the equilibrium and reaction rates of binding chemistry in one condition versus the other. To better understand the differences between 2D and 3D models, we conducted additional simulations varying the height of the simulation space while holding width and length fixed. These simulations were intended to examine the difference between 2D and 3D on a continuum between a purely 2D model and a full 3D model. We varied the height of the simulation box from 5.125 nm (the thickness of a single layer of particles, resulting in a pseudo-2D model that we call the x1 model) to 25.625 nm (five times of the single layer, x5) in increments of 5.125 nm. We note that the thickness here describes the volume in which the center of a particle can move, so the x1 model does allow some diffusion in the height dimension, but too little for particles to pass above or below one another. The width and length of the box is fixed at 50 nm×50 nm. For each test case, we simulated eight *C* values (0.1, 0.15, 0.2, 0.25, 0.3, 0.35, 0.4, 0.45) for fixed reactant concentrations of 0.1 with varying additional inert crowding agent concentrations and varying pure reactant concentrations without inert crowding agents. Figure 1(D) and (E) show simulation snapshots of the quasi-equilibrium state (25 µs) for 0.1*C_R_* at 5.125 nm thickness and 0.1*C_R_*+0.35*C_I_* at 25.625 nm thickness, respectively. Figure 1(F) shows the center position of particles in Figure 1(E), for better visualization. Cyan spheres represent reactant monomers, magenta spheres represent reactant dimers, black spheres represent inert crowding agents, and green spheres represent diffusion limit spheres of SOLM. We allow a diffusion limit sphere can grow beyond the simulation box until the diffusion limit sphere touches the diffusion limit sphere of another neighboring particle. If the center position of a newly sampled particle is outside of the simulation box, then the position of the particle is reflected to the inside of the box based on the reflective boundary assumption in 3DSOLM. In the 5.125 nm height case, shown in figure 1(D), both sampled and reflected positions of a particle can be outside of the simulation box with very low probability, in which case the simulator samples the position of the particle again until the new position of the particle is inside of the box. The hard reflective boundary condition makes the simulation progress fast but the actual simulation volume is extended because it allows a particle to move across the reflective boundary plane until the center position of the particle reach the boundary plane.

**Figure 6:**
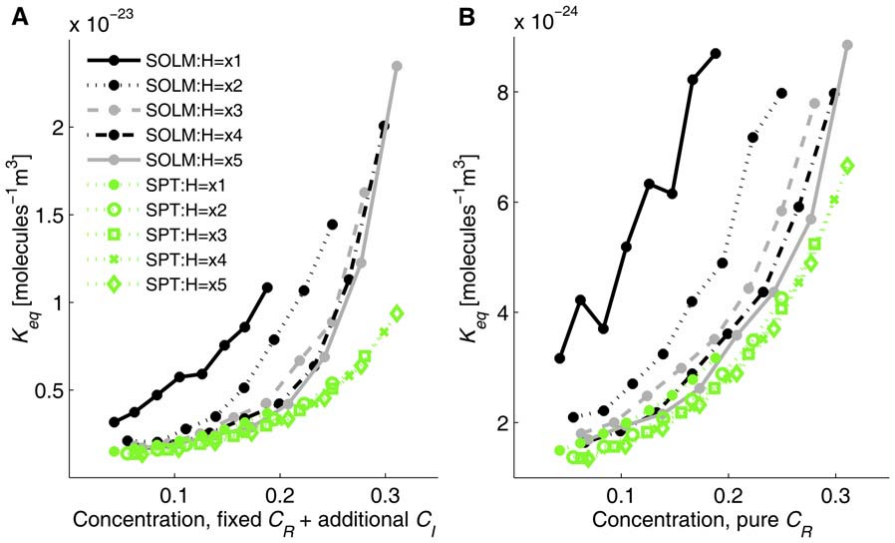
*K_eq_* from SOLM and SPT for variable heights of the simulation space. (A) *K_eq_* from SOLM and SPT for fixed amount of *C_R_*+ additional *C_I_*, (B) *K_eq_* from SOLM and SPT for pure *C_R_* without inert crowding agents. Simulation boxes have length and width of 50 nm in all cases. Five different heights are examined: 5.125 nm (single particle layer with threshold distance, x1), 10.25 nm (double layer, x2), 15.375 nm (x3), 20.5 nm (x4), 25.625 nm (x5). Note that this figure uses concentrations corrected for boundary effects to label the x-axis in contrast to the other figures, in order to better illustrate the trend across concentrations.

For tests varying the height of the simulation box, we used a different convention for labeling the concentration than elsewhere in the manuscript in order to more accurately describe boundary effects. Specifically, we calculated concentrations by accounting for the additional one particle-width beyond the bounding box that part of a particle can occupy. Although this correction was applied throughout the manuscript when calculating excluded volume effects, it is omitted elsewhere in labeling the axes of plots to improve readability. For example, the corrected volume of the single layer case (x1, 5.125 nm) is 55×55×10.125 nm^3^ and the corrected concentration for (*C* = 0.1–0.45) is *C* = 0.043–0.188. Figure 6 shows *K_eq_* curves for both SOLM and SPT for these various height cases using the corrected volume and concentration. As with our previous test cases, *K_eq_* increases as the total concentration increases by the excluded volume effect, and fixed *C_R_* with additional *C_I_* cases show a stronger crowding effect than pure *C_R_* cases. For SOLM, decreasing the height of the simulation box increases the equilibrium constant of the test reaction system, which means that providing less freedom of movement to particles increases the crowding effect similar to the limitation provided by inert crowding agents. Estimated *K_eq_* from SPT, however, cannot distinguish well the effects of varying the height of the simulation box because SPT calculates the non-ideal interaction among particles but does not consider edge effects with the bounding compartment that contains the reactants and inert crowding particles. Although the 1% pure reactant simulations slightly capture the effect of various heights, the estimated *K_eq_* values from SPT do not clearly show the effect of thickness of the simulation box, compared with SOLM.

### Crowding effects over broader ranges of intrinsic reaction rates

**Figure 7:**
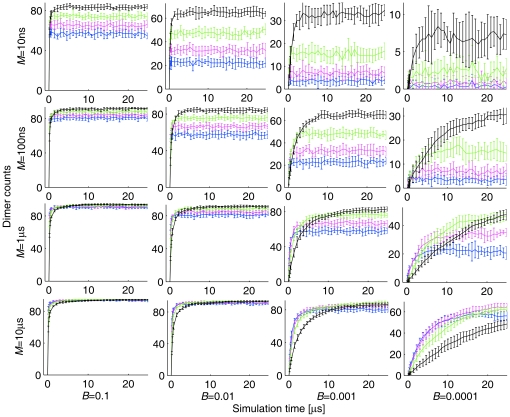
Reaction progress across variations in parameters *B* and *M* at four crowding levels. Blue curves correspond to a concentration of 0.1*C_R_*+0.0*C_I_*, magenta curves to 0.1*C_R_*+0.1*C_I_*, green curves to 0.1*C_R_*+0.2*C_I_*, and black curves to 0.1*C_R_*+0.3*C_I_*. Error bars show the standard deviation of 10 independent runs. Other parameters are set to their default values: *D* = 4.63×10^−11^ m^2^s^−1^, *α* = 2.0, *β* = 1.0, and *d_th_* = 0.125 nm in a 50 nm×50 nm×50 nm simulation box.

**Figure 8:**
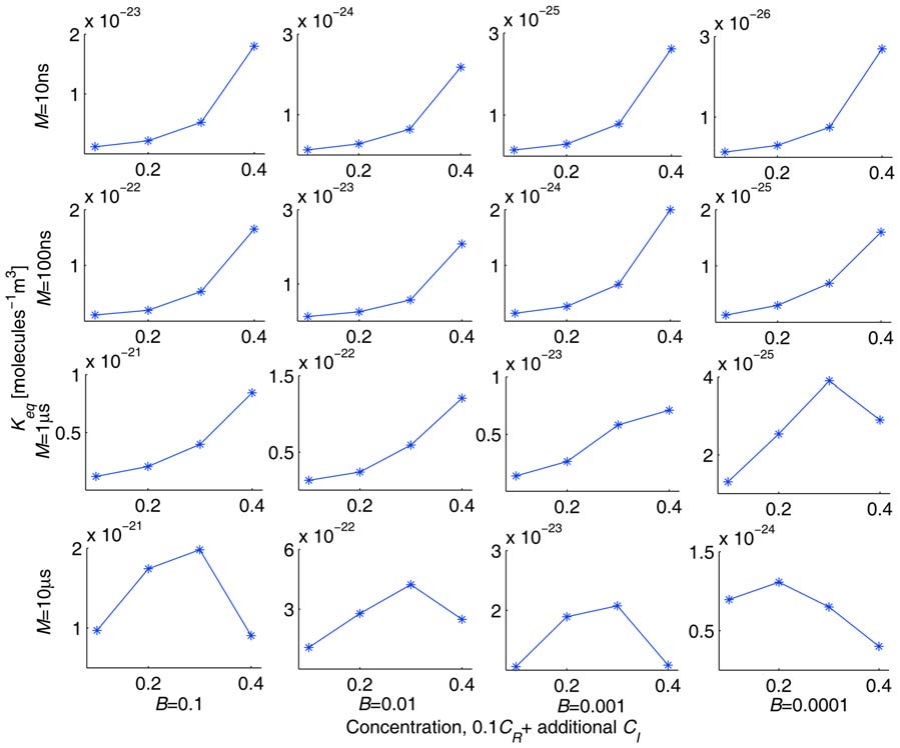
*K_eq_* calculated from SOLM simulations depicted in **figure 7** **.** *K_eq_* values were calculated using average dimer counts from 10 independent runs for 5 time points (5, 10, 15, 20, 25 µs) per run.

Molecular crowding can either enhance or inhibit reaction systems [1], [14] depending on complex interactions among many factors. As a further test of the realism of our model, we have conducted additional validation experiments over much broader parameter ranges to demonstrate the existence of domains in which crowding may enhance, inhibit, or show little effect on binding. We specifically excluded the parameters *α* and *β* shown in our 2D model to be able to modulate the net direction of the crowding effect [30], focusing instead on parameters *B* and *M*, which control the binding probability of collision and the dissociation rate, because these would be expected to interact only indirectly with crowding levels. We simulated homodimerization reactions for parameter variation over four orders of magnitude in *B* (0.1, 0.01, 0.001, 0.0001) and *M* (10 ns, 100 ns, 1 µs, 10 µs) in a 50 nm×50 nm×50 nm simulation box at four crowding levels: 0.1*C_R_*+0.0*C_I_*, 0.1*C_R_*+0.1*C_I_*, 0.1*C_R_*+0.2*C_I_*, and 0.1*C_R_*+0.3*C_I_*. Other parameters are set to their default values (*D* = 4.63×10^−11^ m^2^s^−1^, *α* = 2.0, *β* = 1.0, and *d_th_* = 0.125 nm). We simulated 10 independent runs with 25 µs per run. Figure 7 shows reaction progress curves for these experiments and figure 8 shows inferred equilibrium constants as a function of crowding level for each condition. Figure 7 shows a general trend towards increased dimerization at increased crowding levels, although with considerable variability in crowding influence across conditions. Figure 8 confirms this trend, although it also shows that the effect can be quite variable from one condition to another. In particular, under conditions of slow dissociation (large *M*) high levels of crowding tend to have a net negative effect on binding equilibrium. For example, *K_eq_* for *B* = 0.0001, *M* = 10 ns increases by 20.5 fold from 0.1*C_R_* to 0.1*C_R_*+0.3*C_I_*, while *K_eq_* for *B* = 0.1, *M* = 10 µs decreases by 0.9 fold over the same range of crowding levels. The effects on reaction kinetics of different crowding levels are also quite variable across the parameter space, with figure 7 showing little apparent difference in rates across crowding levels in the presence of high binding probabilities but large variations when collisions rarely lead to binding.

## Discussion

We have built our 3DSOLM model to explore how crowding and other simulation parameters alter the equilibrium state of a model reaction system in 3D, and compared the results with our previous models in 2D. Like the 2D case [16], the 3D model revealed a strong crowding effect, typically enhancing binding affinities by inhibiting dissociation events, across a range of physiologically realistic levels of crowding. This effect was observed in cases of both increasing concentrations of inert crowding agents and increasing reactant concentrations, although it is less pronounced for high reactant concentrations, as would be expected given the greater ability of the pure-reactant system to alter total volume through dimerization. Changes in the parameters *B*, *M*, and *D* in 3DSOLM showed a similar linear variation in binding equilibrium to that seen in 2DSOLM [27]. In addition, changes in the cross-dependent parameters *α* and *C* in 3DSOLM showed a similar nonlinear variation in binding equilibrium to that seen in 2DSOLM [30]. We would expect such effects to be more or less pronounced in different regions of the parameter space and a search across several orders of magnitude does indeed reveals that different parameter domains can lead to very different magnitudes of crowding effects and to either enhancement or suppression of net binding equilibrium. In experimental studies, additional possible interaction types lacking from our model have also been shown to modulate the crowding effect, e.g., the presence of repulsive interactions between particles [2]–[7], attractive interactions between reactants and crowding agents [31], or other nonspecific protein-protein interactions [32]. In other circumstances, crowding has been found to have no strong crowding effect on protein-protein interactions [33]. Our model considers only steric hindrance and the resulting excluded volume effect, and further work would therefore be needed to determine how our conclusions would be affected by the presence of other such long-distance interactions.

Comparison between SOLM and SPT in figure 3 shows that the calculated *K_eq_* values from both methods are close to each other at low-to-moderate crowding levels, but the calculated *K_eq_* values from SOLM are larger than those derived from SPT in high crowding conditions. Although both methods use a hard sphere particle model, SOLM is a particle-based method, simulating individual particles explicitly. SPT, on the other hand, estimates the total excluded volume effect by summing over pairwise contributions, an approximation that can understate the degree to which particle movement is restricted, and thus understate the crowding effect, in conditions of very high crowding [28]. SOLM, conversely, may overstate the crowding effect under similar conditions because of the use of a threshold distance maintained between particles following reaction events. While one can in principle make this threshold distance effect arbitrarily small by reducing the distance, such a reduction would come at a cost of increased run time. The main advantage of SPT is its higher efficiency than SOLM, whose run time increases quadratically with particle counts. Our regression modeling method is intended to give advantages of both, allowing approximations closer to those one would derive from an explicit particle model like SOLM but in run times close to those of a fast analytical approximation like SPT. Because both methods use the hard sphere model and assume that the excluded volume by solvents is negligible, they would be expected to be less realistic than typical Brownian dynamics or molecular dynamics methods, which may explicitly consider effects of water molecules or other nonbonded interactions. The regression approach should, however, in principle be extensible to more realistic particle models such as these.

A key question in this study is how 2D and 3D crowding models differ. The question is relevant in part because of the many 2D studies already in the literature [34], [35] as well as the considerable computational advantages of 2D models over 3D for large systems. In addition, it is important for properly characterizing the crowding effect in genuinely 2D or nearly 2D environments, such as diffusing reaction systems within a membrane. Other examples of systems involving nearly 2D diffusion may include assembly of vesicles and sorting of cargo for intracellular transport [36] and migration of T cells, which can move on the surface of endothelial lining (2D) or interstitial space (3D) [37]. Migration of T cells from 2D to 3D involves specific signaling pathway, such as MEK-Cofilin [37], but it is still unknown how the crowding effect act in this condition. We examined the issue of whether crowding effects are qualitatively different in 2D versus 3D models by interpolating between a cubic 3D space and a pseudo-2D model produced by a simulation space too narrow to allow particles to pass one another in one dimension. Our model does show significant quantitative differences in *K_eq_* as height varies, as shown in figure 6, primarily due to a much stronger barrier to diffusion, once the third dimension is effectively lost. In biological systems, various sizes and shapes of proteins contribute to the crowding effect [38], [39]. Our results suggest that the specific influence of these combinations of shapes and sizes in conjunction with the volumes in which they diffuse must be considered to judge whether a given system is effectively 2D or 3D for the purposes of accurately capturing the crowding effect. The results do, however, suggest that 2D and 3D models provide qualitatively consistent results across various parameters and that these effects do interpolate gradually between the two, indicating that fully 2D models can provide good matches to expected behaviors from nearly 2D systems. Likewise, the results show that the regression approach we developed in 2D as a way of accelerating multiparameter simulations of chemistry in crowded conditions work comparably in 3D as in 2D systems [30]. However, this consistency between 2D and 3D may be lost if we consider additional interactions with compartments or other large obstacles, such as cytoskeleton networks and nondiffusible polymers. Such observations may prove useful in guiding development of efficient crowding models for more complex and more realistic biological systems, and in particular in understanding how one can safely trade off model detail for improved computational tractability without compromising model accuracy.

## Materials and Methods

### Discrete event time calculation

**Figure 9:**
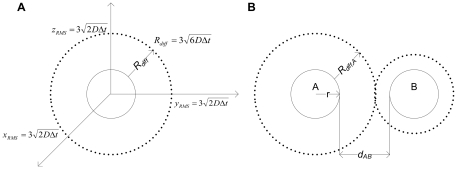
Three dimensional stochastic off-lattice model. (A) The radius of the diffusion limit sphere (*R_diff_*) for a given diffusion coefficient (*D*) and time interval (*Δt*), (B) A discrete event in SOLM.

The main algorithm of 3DSOLM is the same as that described for our prior 2DSOLM model [16]. Both stochastic off-lattice models use the GFRD discrete event simulation method [17] to efficiently simulate particle diffusion in continuous time and space. In 3DSOLM, we apply a hard reflective cubic or rectangular box boundary condition. The test binding reaction system is a homodimerization reaction. All particles in 3DSOLM are spheres. The radius of a diffusion limit sphere (*R_diff_*) in 3DSOLM is set to:
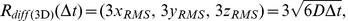
(11)where 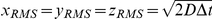 and *D* is diffusion coefficient from the Stokes-Einstein's diffusion equation (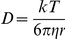 ), shown in figure 9(A). Each Cartesian coordinate of the diffusion limit sphere is three times the standard deviation of the Gaussian distribution for a single coordinate in isolation. The square of the distance in which the particle has diffused in a three-dimensional space can be expressed as the sum of the squares of three independent normal distributions, a quantity that is chi-square distributed with three degrees of freedom. The probability that the particle will be confined to the radius of the diffusion limit sphere (*R_diff_*) is then equivalent to the probability that the chi-square random variable is within (*R_diff_*)^2^, which is 97.07%, covering most of the space of possible Brownian diffusion within the spherical volume. We can calculate the collision time (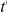) of two diffusion limit spheres as follows:
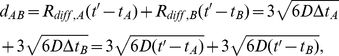
(12)where *d_AB_* is the distance between two particles, *A* and *B*, and *t_A_* and *t_B_* are the times at which the positions of particles *A* and *B* were last determined, shown in figure 9(B). The three different radii of the diffusion limit spheres of a reactant monomer, dimer, and inert particle are then:
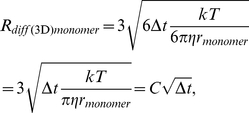
(13)

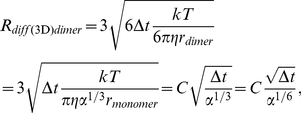
(14)

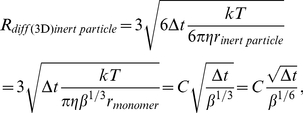
(15)where 
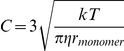
. The parameter 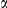 is the ratio of dimer volume to monomer volume (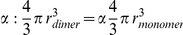), and the dimer radius is therefore 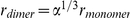. The parameter 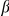 is the ratio of inert particle volume to reactant monomer volume (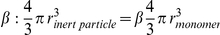), and the inert particle radius is therefore 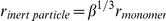.

We can derive a more general equation by plugging Eqs. (13–15) into Eq. (12):
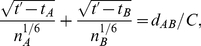
(16)where 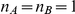, if the particle *A* and *B* are reactant monomers, 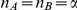, if the particle *A* and *B* are reactant dimers, and 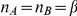, if the particle *A* and *B* are inert particles.

The collision time (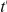) of two diffusion limit spheres, which is the analytical solution of Eq. (16), follows:
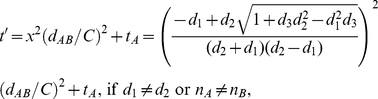


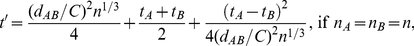
(17)where 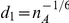, 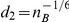, 
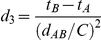
.

### Equilibrium constant calculation by SOLM

We examine the crowding effect for various parameter conditions and different mixtures of reactants and inert crowding agents. To simplify analysis of the crowding effect specifically, we use a simple homodimerization reaction as our test system. The governing chemical equation of the test homodimerization reaction is:
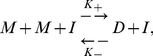
(18)where 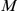 is the reactant monomer, 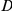 is the reactant dimer, 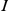 is the inert crowding particle, 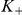 is the forward reaction rate, and 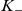 is the reverse reaction rate. The equilibrium constant can be computed from Eq. (18) as follows:
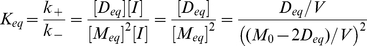


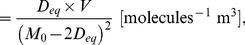
(19)where 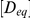 is the concentration of dimers at the quasi-equilibrium state, 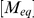 is the concentration of monomers at the quasi-equilibrium state, 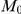 is the number of initial monomers, 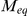 is the number of monomers at the quasi-equilibrium state, 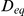 is the number of dimers at the quasi-equilibrium state, and *V* is the volume of simulation space. The concentration of a particle in Eq. (19) is determined by the empirically measured number of particles in the simulation divided by the total volume of the simulation space, similar to standard molar concentrations. The crowding effect of [I] drops out in Eq. (19), because this governing equation is based on the idealized mass-action model. We calculate the estimated *K_eq_* of the binding reaction for various concentrations of [I] using Eq. (19) with the simulation results from 3DSOLM, based on the assumption that 3DSOLM appropriately represents the crowding effect of all particles in the various conditions. In addition, we can estimate the average number of dimers at the quasi-equilibrium state using the estimated *K_eq_*. From Eq. (19), the estimated 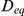 is Eq. (21).
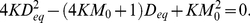
(20)

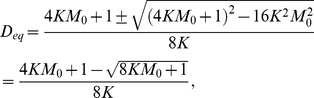
(21)where 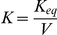.

### Equilibrium constant calculation by SPT

We calculated the apparent equilibrium constant for various densities of reactants and inert crowding particles, and various volume ratio parameter (*α*) values using scaled particle theory [28], [29] and thermodynamic activity theory [14]. In thermodynamic theory, no interaction between particles occurs at the ideal gas state. The equilibrium constant at the ideal state (*K^o^*) is altered by the non-ideal interactions with increasing density of either reactants or inert crowding particles. The non-ideal interaction is approximately calculated using scaled particle theory and activity coefficients of reactants. The apparent equilibrium constant of various densities of particles is 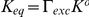 (22), where 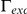 is a correction factor for the excluded volume effect. For our homodimerization reaction in Eq. (18), the correction factor is 
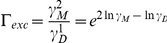
, where 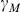 and 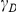 are activity coefficients of reactant monomer and dimer, respectively. Based on scaled particle theory [28], [29], assuming all particles are hard spheres, the activity coefficients for reactant monomers and dimers are
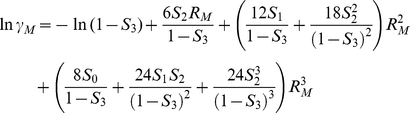


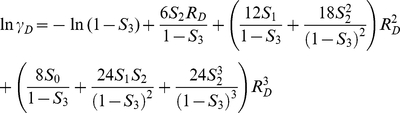


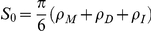


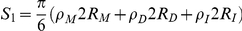


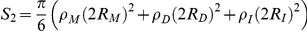


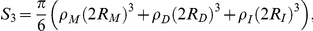
where *ρ* (density) = number of particles/simulation volume and *R_X_* = radius of a particle for each particle species *X*: *M* (reactant monomer), *D*(reactant dimer), or *I*(inert crowding particle). Finally, the apparent equilibrium constant is calculated by multiplying the correction factor by *K^o^*, which is calculated from simulation results at 1% pure reactant concentration for all other given parameter conditions. As shown in table 1, the calculated correction factors to the 1% pure reactant simulations for various parameter conditions were consistently close to 1, which shows that the 1% concentration case is sufficiently dilute to be treated as an ideal state while introducing minimal errors into subsequent SPT estimations.

### Simulation conditions and experiments

3DSOLM has seven different parameters: the total concentration (*C*), defined as the volume ratio of all particles to the investigated simulation space; the probability of binding upon a collision between two reactant monomers (*B*); the mean time for dissociation events (*M*), defined as the inverse of the dissociation rate constant; the diffusion coefficient (*D*); the volume ratio of a dimer to a reactant monomer (*α*); the volume ratio of an inert particle to a reactant monomer (*β*); and the threshold distance between two particles (*d_th_*), describing the maximum distance at which two particles can interact with one another. We established a baseline simulation parameter set with default parameter values of *B* = 0.7, *M* = 1 ns, *D* = 4.63×10^−11^ m^2^s^−1^, *α* = 2, *β*  = 1, and *d_th_* = 0.125 nm. These default values were chosen based on our prior 2DSOLM simulation studies [16], [27], [30] to produce a reasonably strong crowding effect as well as to approximate a reasonable range of temperature and viscosity conditions of the cytoplasm [40], [41]. The radius of a reactant monomer is fixed at 2.5 nm. Initially, all reactants are monomers for the test homodimerization reaction. To achieve the maximum possible packing density, however, we placed particles initially on the hexagonal close-packed spherical lattice at the maximum possible packing density for whichever of reactant monomers and crowding agents occupies the larger total volume and then randomly inserted particles into the corresponding grid positions. The radius of the spherical lattice is the radius of selected particles for maximum density plus half of the threshold distance, in order to prevent particles from interacting with each other in the initial state. This protocol was developed because it makes it possible to initialize in highly crowded conditions where independent uniform placement of particles would usually result in overlapping particles. Initially, all particles are located inside of the simulation box. The reflective boundary condition in 3DSOLM allows a particle to move partially outside the simulation space until the center position crosses the simulation boundary plane, similar to 2DSOLM [16]. Because a particle in 3DSOLM can partially cross the boundary plane of the simulation box after the initial state, we corrected the total concentration values to account for the additional volume outside the simulation box that particles can partially occupy. Each simulation was run for 25 µs with 10 repetitions per simulation, with progress recorded every 0.15625 µs. For each condition, we measured reaction progress by the mean number of dimers as a function of time across all simulations.

The 3DSOLM simulation program was implemented in C++ and run on a Linux Beowulf cluster. The collected data files were analyzed and plotted using Matlab (R2008a).

### Simulation movie file

We created a movie file to demonstrate the simulation process in 3DSOLM and show the effect of molecular crowding. Video S1 presents a comparison of 0.1 *C_R_* and 0.1 *C_R_*+0.35 *C_I_* simulations. The first half of the movie shows each system in the initial (pre-equilibration) state, and the second half of the movie shows a quasi-equilibrium state. High-resolution versions of the movies can be downloaded from: http://www.cs.cmu.edu/~russells/projects/crowding/SOLM.html.

## Supporting Information

Video S1 **Simulation movie of 3DSOLM in low and high crowding conditions.** The movie shows sample trajectories for two simulations, the left side representing a low crowding (0.1 *C_R_*) case and the right side representing a high crowding (0.1 *C_R_*+0.35 *C_I_*) case. All other parameters are set to their default values: *B* = 0.7, *M* = 1.0 ns, *D* = 4.6×10^−11^ m^2^s^−1^, α = 2.0, β = 1.0, *d_th_* = 0.125 nm in a 50 nm×50 nm×50 nm simulation box. The first half of the movie shows each system in its initial pre-equilibration state and the second half of the movie shows the same systems in a quasi-equilibrium state. Cyan spheres are reactant monomers, magenta spheres are reactant dimers, and black spheres are inert crowding particles. Diffusion limit spheres are shown in green for all particles.
(MOV)
